# Targeted Therapy of Interleukin-34 as a Promising Approach to Overcome Cancer Therapy Resistance

**DOI:** 10.3390/cancers15030971

**Published:** 2023-02-03

**Authors:** Giovanni Monteleone, Eleonora Franzè, Claudia Maresca, Marco Colella, Teresa Pacifico, Carmine Stolfi

**Affiliations:** 1 Department of Systems Medicine, University of Rome “Tor Vergata”, 00133 Rome, Italy; 2 Gastroenterology Unit, Policlinico Universitario Tor Vergata, 00133 Rome, Italy

**Keywords:** immunotherapy, chemotherapy, CSF1R, tumor-associated macrophages, myeloid-derived suppressor cells

## Abstract

**Simple Summary:**

In the last decades, identification of the factors/mechanisms leading to cancer development has advanced the way clinicians combat this pathology. Indeed, the use of adjuvant chemotherapies and targeted therapies has markedly contributed to prolonging the survival of cancer patients. Unfortunately, however, many cancer patients experience primary or acquired resistance to these therapies, and this has been linked to various factors, including the presence of a tumor microenvironment that restrains the anti-tumor immunity. In this article, we describe the ability of interleukin-34, a protein produced in excess in many cancers, to modulate the function of various immune cells, with the downstream effect of generating a tumor microenvironment that sustains cancer cell growth and, at the same time, enhances the resistance of cancers against chemotherapy and immunotherapy.

**Abstract:**

Chemotherapy and immunotherapy have markedly improved the management of several malignancies. However, not all cancer patients respond primarily to such therapies, and others can become resistant during treatment. Thus, identification of the factors/mechanisms underlying cancer resistance to such treatments could help develop novel effective therapeutic compounds. Tumor-associated macrophages (TAMs), myeloid-derived suppressor cells (MDSCs), and regulatory T cells (Tregs) are major components of the suppressive tumor microenvironment and are critical drivers of immunosuppression, creating a tumor-promoting and drug-resistant niche. In this regard, therapeutic strategies to tackle immunosuppressive cells are an interesting option to increase anti-tumor immune responses and overcome the occurrence of drug resistance. Accumulating evidence indicates that interleukin-34 (IL-34), a cytokine produced by cancer cells, and/or TAMs act as a linker between induction of a tumor-associated immunosuppressive microenvironment and drug resistance. In this article, we review the current data supporting the role of IL-34 in the differentiation/function of immune suppressive cells and, hence, in the mechanisms leading to therapeutic resistance in various cancers.

## 1. Introduction

Cancer is one of the major public health problems worldwide. It has been estimated that in 2022, nearly 2 million new cancer cases and more than half a million cancer deaths could occur in the United States [1]. However, it is noteworthy that the 5-year relative survival rate for all cancers combined has increased continuously in the last 3 decades, because of the better understanding of the factors/mechanisms driving cancer development. Hence, reduced exposure of the host to pro-carcinogenic insults (e.g., smoking), better surgical techniques, and accurate screening programs (e.g., mammography for women aged 45 to 74 for breast cancer; quantitative fecal immunochemical testing and colonoscopy for individuals between 50 and 74 years for colorectal cancer; prostate-specific antigen (PSA) testing for prostate cancer; esophagogastroduodenoscopy and the *Helicobacter pylori* test in regions with high gastric cancer incidence and mortality; the blood test for the CA 125 tumor marker in combination with ultrasound for women with a “high-risk” family history of ovarian cancer; and abdominal ultrasonography in combination with biomarkers, such as α-fetoprotein, in patients with a “high risk” of developing hepatocellular carcinoma (HCC), such as those with cirrhosis, including patients with hepatitis B virus (HBV) and hepatitis C virus (HCV) infections, as well as with nonalcoholic fatty liver disease) allow early detection of neoplastic lesions [1].

The decline in cancer death rate relies also on improvements in treatment protocols, including adjuvant chemotherapies and targeted therapies. For instance, the use of tyrosine-kinase inhibitors has markedly increased the 5-year relative survival rate of patients with chronic myeloid leukemia. Indeed, analysis of studies conducted between 2000 and 2012 showed that, for the whole population of patients with chronic myeloid leukemia in the chronic phase, the 5-year survival was only slightly lower than that of the matched general population [2]. More recently, immunotherapy (i.e., antibodies blocking programmed cell death protein 1 (PD-1) and/or cytotoxic T-lymphocyte-associated protein-4 (CTLA-4)) has changed the landscape of systemic therapy for metastatic solid tumors (e.g., metastatic melanoma, non-small-cell lung cancer, and genitourinary cancers), thereby improving the survival of cancer patients who were resistant to traditional therapies [3,4,5,6]. Concerning CTLA-4 immune checkpoint inhibitors, in addition to the fully human monoclonal IgG1 antibody ipilimumab (Yervoy), approved in March 2011 and currently used for the treatment of several cancers (e.g., melanoma, renal cell carcinoma (RCC), microsatellite instability-high (MSI-H) or mismatch repair deficient (dMMR) metastatic colorectal cancer (CRC), HCC, and non-small cell lung cancer (NSCLC)) [7], the Food and Drug Administration (FDA) has recently approved tremelimumab (Imjudo), a fully human monoclonal IgG2 antibody, as part of a first-line immunotherapy combination with the PD-L1 blocker durvalumab for non-resectable HCC [8].

To date, the FDA has approved three anti-PD-1 antibodies: pembrolizumab (Keytruda), nivolumab (Opdivo), and cemiplimab (Libtayo) [9]. Pembrolizumab, a humanized IgG4 antibody, was initially approved in September 2014, following the results of the KEYNOTE-001 clinical trial (clinicaltrials.gov/NCT01295827) studying patients with unresectable or metastatic melanoma and patients with NSCLC. Several additional indications without biomarker requirements have been approved since then, including indications for the treatment of patients with recurrent or metastatic cutaneous squamous cell carcinoma, adult and pediatric patients with refractory classical Hodgkin’s lymphoma, individuals with advanced RCC, mediastinal large B cell lymphoma, and HCC, locally advanced or metastatic urothelial carcinoma for patients who are not eligible for chemotherapy containing cisplatin or who have had disease progression during or after platinum-containing chemotherapy [9].

Nivolumab, a fully human IgG4 monoclonal antibody, was approved following the pivotal trial CheckMate-037, in December 2014, for the treatment of patients with non-resectable metastatic melanoma who have experienced disease progression following ipilimumab and, if *BRAFV600* mutation positive, a BRAF inhibitor [10]. Since then, new indications have been approved, such as for treating patients with metastatic NSCLC with progression on or after platinum-based chemotherapy; for the treatment, either alone or in combination with ipilimumab, of patients with unresectable/metastatic melanoma; for treating individuals with advanced RCC, classical Hodgkin’s lymphoma, recurrent or metastatic squamous cell carcinoma of the head and neck (HNSCC), locally advanced or metastatic urothelial carcinoma, HCC, or esophageal squamous cell carcinoma (ESCC); and for patients with unresectable malignant pleural mesothelioma [9].

Cemiplimab (Libtayo), a human IgG4 monoclonal antibody, was approved in 2018 for treating patients with metastatic cutaneous squamous cell carcinoma (CSCC) or locally advanced CSCC who are not candidates for curative surgery or radiation [11], and later for the treatment of NSCLC [9].

These approaches are based upon the demonstration that many cancers promote dysregulation of immune-checkpoint proteins (e.g., PD-1), which are physiologically crucial for limiting abnormal T-cell-driven immune responses [12]. Unlike many antibodies used for cancer therapy, immune checkpoint blockers do not target cancer cells directly; instead, they target lymphocyte receptors or their ligands, with the downstream effect of enhancing T cell-dependent antitumor activity [13,14]. 

Unfortunately, however, many cancer patients experience primary (existing before treatment) or acquired (generated after therapy) resistance to these drugs [15].

## 2. Mechanisms of Cancer Resistance to Standard Therapy and Immunotherapy

Drug resistance in cancer is a well-known phenomenon that occurs when cancer cells are, or become, tolerant to pharmaceutical treatment. Resistance to anticancer drugs is caused by a variety of factors, including genetic mutations and/or epigenetic changes, conserved but overregulated drug efflux, and various other cellular and molecular mechanisms.

Primary resistance can be caused by: (i) pre-existing (hereditary) mutations resulting in decreased responsiveness of cancer cells to both chemo- and immunotherapy (e.g., lack of the estrogen receptor (ER), progesterone receptor (PR), and amplification of HER-2/Neu in triple negative breast cancer cells [16] and HER2 overexpression resulting in a poorer outcome of cisplatin treatment in gastric cancer patients [17]); (ii) tumor heterogeneity in which pre-existing insensitive subpopulations, including cancer stem cells, will be selected after drug treatment, thus leading to relapse in later stages of the therapeutic regimen; and (iii) activation of intrinsic pathways used to defend the cell against environmental toxins (in this case, against anticancer drugs) (e.g., ATP binding cassette (ABC) transporter-mediated drug efflux [18] and glutathione (GSH)/glutathione S-transferase system [19] working to reduce cellular drug accumulation or detoxify drug treated cancer cells, respectively] 

Acquired resistance can be identified by a gradual reduction in the anticancer efficacy of a given drug after treatment. Acquired resistance can be the result of: (i) activation of a second proto-oncogene that becomes the new driver gene; (ii) mutations or modified expression levels of the drug targets; and (iii) dynamic changes in the tumor microenvironment (TME)—that is, the surrounding space composed of immune cells, stroma, and vasculature—in the course of treatment [20].

With regard to the latter point, TME can mediate resistance to anticancer drugs through several mechanisms, including preventing immune clearance of tumor cells, inhibiting drug absorption, and stimulating paracrine growth factors to signal cancer cell growth [20]. For example, blockade of an immune-checkpoint protein can induce tumor regression only when there is a pre-existing antitumor immune response to be ‘unlocked’ when the pathway is blocked. In contrast, the presence of an immunosuppressive microenvironment, which inhibits intratumoral immune responses, can eventually hamper the efficacy of immunotherapy [21]. Recent studies have also shown that activation of the immune system is one of the mechanisms underlying the benefit of cytotoxic chemotherapy. Indeed, several chemotherapeutics trigger cancer cell death, thus promoting the release of a variety of factors that boost robust anti-tumor immune responses [22,23]. These discoveries have provided the rationale for many studies in cancer patients combining immune agents with chemotherapy. On the other hand, there is evidence that chemotherapy-treated cancer cells can release molecules, which in turn promote differentiation and activation of tumor-associated macrophages (TAMs) and myeloid-derived suppressor cells (MDSCs), thereby contributing to generating a tumor microenvironment that restrains the anti-tumor immunity [24].

One of such molecules is interleukin-34 (IL-34), a cytokine produced by cancer cells, TAMs, and stromal cells, which acts as a linker between induction of a tumor-associated immunosuppressive microenvironment and chemo/immunotherapy resistance.

In this article, we review the available evidence supporting the role of IL-34 in promoting cancer resistance to chemotherapy and immunotherapy.

## 3. Expression of IL-34 and IL-34 Receptors in Cancer

The functions of IL-34 are mediated by three distinct receptors or co-receptors, namely, CSF1R (CD115), CD138 (syndecan-1), and protein-tyrosine phosphatase zeta (PTP-ζ) [25,26,27,28]. Since its discovery, IL-34 has been indicated as a growth factor for monocytes, and it is now evident that IL-34 effects on monocyte viability occur through a mechanism independent of colony-stimulating factor 1 (CSF1), also known as the macrophage CSF (M-CSF), another ligand of CSF1R [25]. IL-34 and CSF1 bind to overlapping regions of CSF1R, but the two ligands can trigger distinct intracellular pathways, likely as a result of the different stability of the interaction of each cytokine with CSF1R [26,29,30,31]. This would explain the non-redundant functions of IL-34 and CSF1 on the survival, proliferation, and differentiation of monocytes/macrophages [29]. The differences between the two cytokines in modulating monocyte/macrophage functions could also be linked to the fact that, unlike IL-34, CSF1 does not interact with CD138 and PTP-ζ [27,28].

**Figure 1:**
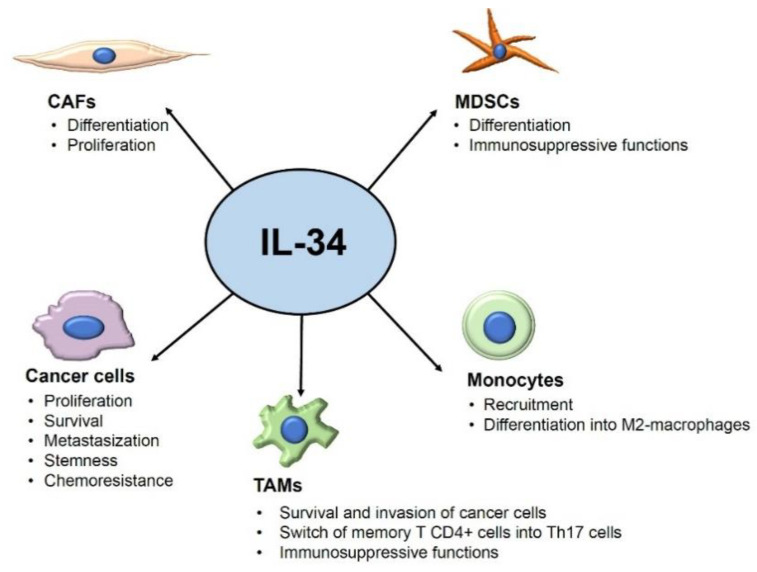
Overview of the tumor-promoting effects of IL-34 on its target cells. Arrows indicate the targets of IL-34 and the IL-34-driven tumor effects. Abbreviations: CAFs: cancer-associated fibroblasts; MDSCs: myeloid-derived suppressor cells; TAMs: tumor-associated macrophages.

In physiological conditions, IL-34 RNA is expressed by many organs and tissues, but its protein levels are more pronounced in lymphoid tissues (i.e., spleen), the brain, and the skin [32,33,34]. In addition to monocytes/macrophages, many other cells produce IL-34 (e.g., neurons, peritubular cells, suprabasal keratinocytes, theca cells, Leydig cells, and fibroblasts) [35,36,37,38]. Recent studies have shown that IL-34 production is deregulated in many infectious, inflammatory, and neoplastic disorders, and they unveiled the contribution of the cytokine to several pathological states [39,40,41]. One of the first studies documenting the over-expression of IL-34 in neoplastic diseases was provided by Baud’huin and colleagues, who reported elevated levels of the cytokine in giant cell tumor (a benign bone tumor) and documented the ability of IL-34 to stimulate osteoclastogenesis via a CSF1-independent CSF1R activation [42]. Afterward, many authors have contributed to delineating the role of IL-34 in the control of the function of several cell types that sustain cancer initiation and progression (Figure 1).

IL-34 is overexpressed by cancers developing in the alimentary tract, such as esophageal squamous, gastric cancer, and CRC, as well as by ovarian, lung, hepatocellular, and pancreatic cancers, and metastatic melanoma (Table 1).

IL-34 is also highly expressed in triple-negative breast cancer (TNBC), which is characterized by the lack of therapeutic receptors, epidermal growth factor-related 2, progesterone receptor, and estrogen receptor on the cell surface [61]. Although further work is needed to clarify the exact contribution of IL-34 in the initiation and progression of each of these malignancies, the overall evidence indicates that the expression of IL-34 in such tumors correlates with their metastatic behavior and poor survival of the patients [61]. This is in line with experimentation in mouse models of tumorigenesis showing a key role of IL-34 in promoting cancer metastasis [62].

## 4. IL-34 Contributes to Generating a Tumor Microenvironment That Restrains the Anti-Tumor Immunity

TAMs constitute the dominant immune cell population in various tumors. TAMs can be functionally divided into two main subtypes (M1-like and M2-like macrophages, respectively), depending on the profile of the molecules synthesized and, hence, their ability to either restrain or promote neoplastic growth and progression [63]. M1-like macrophages are pro-inflammatory and trigger robust T cell- and natural killer cell-mediated anti-tumoral responses, whereas alternatively activated, anti-inflammatory M2-like macrophages contribute to tissue remodeling and stimulate cancer cell growth, invasion, angiogenesis, and tumor metastasis [64]. Moreover, M2-type TAMs play a critical role in the therapeutic resistance of cancers, and increased infiltration of these cells into tumors following chemotherapy represents a hallmark of developing chemoresistance and correlates with poor clinical outcomes [65,66]. Therefore, in recent years, enormous efforts have been made to ascertain factors/mechanisms that promote the differentiation and function of M2-type TAMs, with the ultimate goal to identify new therapeutic targets to overcome chemoresistance. Accumulating evidence suggests that IL-34 could be one of such targets. Support for this hypothesis comes from studies in lung cancer showing that lung cancer cells exposed to chemotherapeutic agents exhibit enhanced NF-kB activation, which in turn sustains elevated production of IL-34 in chemoresistant cells [67]. These findings are in line with the demonstration that chemotherapy-driven NF-kB activation promotes cancer chemoresistance, mainly via the induction of anti-apoptotic genes [68]. Chemoresistant lung cancer cell-derived IL-34 activates an autocrine pathway, which enhances the survival of neoplastic cells, but at the same time, promotes the in vitro differentiation of monocyte-derived M2-polarized macrophages and enhances the immunosuppressive function of TAMs through a C/EBPβ-mediated mechanism [67]. Consistently, studies in a humanized mouse model of lung cancer showed that IL-34-producing chemoresistant tumors exhibit increased numbers of M2-type TAMs and reduced frequencies of tumor-infiltrating cytotoxic CD8^+^ T cells [67]. Along the same line are the results published by Nakajima and colleagues, who showed that neoadjuvant chemotherapy upregulates the induction of IL-34, but not CSF1, on esophageal squamous cell carcinoma cells. It was also shown that expression of IL-34 was more pronounced in patients not responsive to neoadjuvant chemotherapy than in those who were responsive, and patients with cancers expressing high levels of IL-34 had worse prognoses as compared with patients with IL34-low carcinoma [69]. Furthermore, human esophageal squamous cell carcinoma cell lines treated with 5-fluorouracil/cisplatin expressed high IL-34 and promoted induction of CD163, a marker of M2-type TAMs, in human peripheral blood monocytes [69]. Studies in ovarian cancer confirmed the induction of IL-34 in cancer cells by cytotoxic chemotherapy and the reduced overall survival of patients with high IL-34 expression. Moreover, in a mouse model of ovarian cancer, lack of IL-34 attenuated tumor progression, and this finding was associated with reduced increased infiltration of the tumors with T lymphocytes [49]. In CRC tissues, there is a positive correlation between IL-34 and CD163, and CRC-infiltrating immune cells respond to IL-34 by up-regulating M2-type macrophage-related markers [44]. In the MC38 CRC murine model, selective inhibition of CSF1R reduces the number of CD163^+^ macrophages [70] and increases the number of cytotoxic CD8^+^ T-cells within the tumor, thereby delaying tumor growth [71]. MDSCs, a heterogeneous population of immature myeloid cells, are generated in the bone marrow and can terminally differentiate into mature granulocytes, dendritic cells, or macrophages. In pathological conditions, such as cancer, the differentiation of precursor cells is partially blocked, thereby causing the accumulation of immature myeloid cells, defined as MDSCs [72]. Like TAMs, MDSCs constitute an important component of the immunosuppressive tumor microenvironment, and their infiltration into tumors is frequently associated with drug resistance and correlates with poor prognosis. MDSCs promote tumor immune escape via multiple mechanisms, including the production of immunosuppressive cytokines, expression of regulatory factors (e.g., arginase and indoleamine 2,3-dioxygenase), and interaction with and stimulation of other regulatory cells (e.g., Tregs) [73]. In cancer, MDSCs are relevant not only for promoting immunosuppression, but also for enhancing angiogenesis and tumor progression [73,74]. The mechanism of MDSCs-mediated chemoresistance acquisition in cancer is not well known, but recent studies support the contribution of IL-34 in the modulation of MDSCs differentiation and function. Kajihara and colleagues showed that TNBC-derived IL-34 induced differentiation of myeloid stem cells into monocytic MDSCs (M-MDSCs), which recruited Tregs and contributed to the creation of an immunosuppressive microenvironment [75]. At the same time, IL-34 decreased the differentiation of myeloid stem cells into polymorphonuclear MDSCs, thus leading to an acquisition of resistance to chemotherapy. The latter finding seems to rely on the suppression of angiogenesis as a blockade of IL-34 in mice with established tumor induced by the TNBC cell line 4T1, leading to a dramatic increase in vasculature, thereby restoring the penetration of chemotherapeutic agents and tumor sensitivity to paclitaxel (PTX), one of the standard treatments of TNBC. Importantly, analysis of the immune cells infiltrating the tumor showed that combination therapy with PTX and IL-34 blocker was superior to PTX monotherapy in reducing the fraction of M-MDSCs and increasing the number of T cells [75].

Although there is little evidence about the role of IL-34 in the differentiation and action of Tregs in cancer, studies in other systems demonstrated the involvement of IL-34 in human and rat Treg-mediated suppressive functions, as well as the ability of IL-34 to induce in vivo and in vitro CD4^+^ and CD8^+^ Tregs through monocytes polarization toward M2-type macrophages [76,77].

Altogether, these data suggest that the pro-tumorigenic action of IL-34 is, at least in part, mediated by the induction of immunosuppressive TAMs.

## 5. IL-34 and Cancer Immunotherapy

As pointed out above, the blockade of PD-1 and/or CTLA-4 enhances anti-tumor T cell-dependent immune response. However, such a therapeutic strategy does not always associate with benefits in cancer patients due to tumor-intrinsic or -extrinsic mechanisms for escaping immune surveillance [78]. In this regard, an abundance of M2-type TAMs, MDSCs, and Tregs in the tumor tissue contributes to immunotherapy resistance [79,80,81]. Preliminary evidence suggests that IL-34 signaling could make a contribution to cancer resistance to immunotherapy. Han and colleagues described a clinical case of a patient with metastatic refractory melanoma that acquired resistance to anti-PD-1 immunotherapy and exhibited enhanced expression of IL-34 in refractory melanoma tissues [82]. Studies conducted by Hama and colleagues showed that BALB/c mice inoculated with IL-34-expressing CT26 cells developed tumors resistant to anti-PD-1 antibody treatment. In the same model, combination therapy with anti-PD-1 and anti-CTLA-4 induced substantial tumor growth inhibition, which was further enhanced by anti-IL-34 treatment. Consistently, tumors generated by inoculation of mice with IL-34-deficient CT26 cells exhibited a good response to treatment with anti-PD-1 antibody [83]. The same group transplanted human lung cancer tissue expressing both IL-34 and PD-L1 in immunologically humanized mice to determine the effect of IL-34 neutralization, along with the immune checkpoint blockade, in human tumors. Combination therapy was superior to monotherapy in reducing tumor growth [84]. Indirect evidence comes from studies by Shi and colleagues, who treated mice bearing the CT26 and MC38 colon tumors with a combination of PLX3397 (an oral inhibitor of MCSF1R), anti-PD-1 antibody, and oncolytic viruses (OVs), which promote tumor T-cell infiltration due to their ability to selectively infect and kill tumor cells [85]. Combined treatment enhanced the number of T cells in the tumor and augmented anti-tumor CD8^+^ T-cell function, thereby conferring tumor control and prolonged survival of mice. In particular, mice treated with PLX3397 exhibited reduced numbers of M2-type TAMs and enhanced response to anti-PD-1 therapy [85]. These observations are in line with findings of other studies showing that the limited efficacy of PD-1 and CTLA-4 antagonists, when used as single agents to restrain cancer growth, can be enhanced by MCSF1R blockers [86] Although these latter findings do not exclude the possibility that some of the benefit seen in animals receiving MCSF1R blockers are due to the inhibition of CSF1 function, the overall data would seem to indicate that IL-34 can target several regulatory cell types within the tumor and favors an anti-inflammatory environment, with the downstream effect of interfering with immunotherapy.

## 6. Conclusions

Accumulating evidence indicates that IL-34 is produced in many cancers and supports the hypothesis that this cytokine triggers multiple signals that enhance cancer cell growth and diffusion. Nonetheless, it is worth noting that some studies suggest a beneficial role for IL-34 in particular tumor contexts [87,88,89], thus raising the question of whether the role of IL-34 in cancer is dependent on tumor type, location, or even treatment regimens.

In some cancers, IL-34 production can be further increased by chemotherapies, and this is particularly evident in those patients who are, or become, resistant to chemotherapy and/or immunotherapy. The findings described here also indicate that IL-34 facilitates the differentiation of immunosuppressive cells within the tumor microenvironment, leading to therapeutic resistance and poor outcomes in various cancers. Therefore, the possibility to use inhibitors of IL-34 signaling to overcome cancer resistance could open up a promising opportunity for the treatment of such patients. However, before moving into the clinic, further pre-clinical work is needed to better clarify if IL-34-mediated cancer resistance to therapies is either a general phenomenon occurring in all the patients who do not respond to such treatments or restricted to specific subsets of cancers/patients. Experimentation will be also needed to understand if the circulating levels of IL-34 may contribute to the early identification of non-responders and whether the positive effect of IL-34 on the induction of the immunosuppressive cells is shared with and/or enhanced by CSF1. If this is the case, blockade of CSF1R, rather than IL-34 alone, could be more appropriate to overcome resistance to chemotherapy/immunotherapy.

## Figures and Tables

**Table 1:** Expression and function of IL-34 in various human cancers.

Cancer	System	IL-34 Functions	References
Colorectal	Cancer cell line	-Increases cell proliferation and invasion -Enhances resistance to oxaliplatin-induced death	[43]
	TAMs	-Induces type 2 macrophage markers -Enhances IL-6 production	[44]
	CAFs	-Promotes CAFs differentiation and proliferation	[45]
Breast	TAMs	-Enhances proliferation, Chemotaxis, and tumor infiltration	[46]
	Cancer cells	-Enhances metastatic properties	[47]
Ovarian	Macrophages and TAMs	-Promotes the switch of non-Th17 committed memory T cells into conventional Th17 cells	[48]
	Cancer cells	-Promotes survival of chemoresistant cancer cells	[49]
	TAMs	-Enhances the tumorigenic and immunosuppressive functions	[50]
Hepatocellular carcinoma	Cancer cell line	-Increases growth and metastatic properties	[51]
	TAMs	-Increases TGF-β production	[51]
Hepatoblastoma	Cancer cell line	-Increases cell growth and chemoresistance	[52]
	TAMs	-Promotes M2 polarization -Stimulates production of IL-6 -Enhances chemotaxis and tumor infiltration	[52]
Cholangiocarcinoma	TAMs	-Induces differentiation and activation and promotes tumor infiltration	[53]
	Cancer stem cells	-Promotes stemness features	[53]
Osteosarcoma	Cancer cells	-Induces cell proliferation and metastasis	[54]
	TAMs	-Increases recruitment into tumor tissue	[54]
	Endothelial cells	-Stimulates proliferation and vascular cord formation	[54]
Multiple myeloma	CD141^+^Monocytes	-Accelerates multiple myeloma-induced osteoclast formation	[55]
Castration-resistant prostate cancer	TAMs	-Induces differentiation and chemotaxis and tumor infiltration	[56]
Adult T-cell leukemia/lymphoma	Cancer cell line	-Increases proliferation	[57]
Gastric cancer	Cancer cells line	-Increases proliferation, clone formation, migration, and invasion	[58]
Acute monocytic leukemia	Cancer cell line	-Increases proliferation and colony formation	[59]
Pancreatic ductal adenocarcinoma	Portal blood MDSCs	-Promotes differentiation and immune suppressive functions	[60]

Abbreviations: TAMs: tumor-associated macrophages; CAFs: cancer-associated fibroblasts; MDSCs: myeloid-derived suppressor cells.
